# Treatment plan quality comparison between ZAP‐Axon^®^ Krylov optimizer and Leksell Gamma Knife^®^ Lightning optimizer for brain metastasis

**DOI:** 10.1002/acm2.70851

**Published:** 2026-11-01

**Authors:** Yongsook C. Lee, Alexander Maslowski, Rupesh Kotecha, Ranjini Tolakanahalli, D Jay Wieczorek, Matthew D. Hall, Minesh P. Mehta, Michael W. McDermott, Alonso N. Gutierrez

**Affiliations:** ^1^ Department of Radiation Oncology Herbert Wertheim Cancer Institute Baptist Health South Florida Miami Florida USA; ^2^ Department of Oncological Sciences Herbert Wertheim College of Medicine Florida International University Miami Florida USA; ^3^ ZAP Surgical Systems, Inc. San Carlos California USA; ^4^ Department of Translational Medicine Herbert Wertheim College of Medicine Florida International University Miami Florida USA; ^5^ Department of Neurosurgery Miami Neuroscience Institute Baptist Health South Florida Miami Florida USA

**Keywords:** brain metastasis, Krylov, Leksell Gamma Knife, Lightning, stereotactic radiosurgery, ZAP‐Axon

## Abstract

**Purpose:**

This study compares stereotactic radiosurgery (SRS) treatment plan quality between the recently launched ZAP‐Axon^®^ Krylov optimizer and the Leksell Gamma Knife^®^ (LGK) Lightning optimizer for brain metastasis (BM).

**Methods:**

Thirty (*n* = 30) patients who were previously treated with LGK SRS or fractionated SRS were selected and divided into three groups: large post‐operative resection cavities of BM (BM_lg, postop_), large intact BM (BM_lg, intact_) and small intact BM (BM_sm, intact_). Based on our institutional planning guidelines, the 30 clinical cases were replanned using ZAP‐Axon Krylov and LGK Lightning optimizers without manual adjustments. Statistical analysis was performed to compare plan quality metrics (Paddick conformity index [PCI], gradient index [GI], target coverage [TC], prescribed isodose line [Rx IDL], and treatment time/scaled beam‐on‐time [sBOT] [min]) between the two optimizers.

**Results:**

For the BM_lg, postop_ group, mean values of PCI, GI, TC, Rx IDL, and treatment time/sBOT (min) for Krylov versus Lightning plans were 0.91 versus 0.91, 2.48 versus 2.56, 99.5% versus 99.7%, 51.1% versus 53.7% and 37.4 min versus 36.5 min, respectively. All metrics except for the Rx IDL (%) were not statistically significantly different. For the BM_lg, intact_ group, corresponding values were 0.93 versus 0.93, 2.42 versus 2.57, 99.5% versus 99.7%. 51.5% versus 53.0% and 35.9 min versus 36.3 min. While GI was statistically significantly different (*p* <0.05) between the two optimizers, the rest of the metrics were not. For the BM_sm, intact_ group, corresponding values were 0.71 versus 0.64, 3.10 versus 3.26, 99.5% versus 99.5%, 61.9% versus 53.0% and 8.1 min versus 19.8 min. In this group, PCI and treatment time/sBOT were statistically significantly different (*p* <0.05), whereas GI and Rx IDL were not.

**Conclusion:**

Without manual adjustments, ZAP‐Axon Krylov plans were comparable to LGK Lightning plans for large BM lesions, but the Krylov optimizer outperformed the Lightning optimizer for small BM lesions.

## 1 INTRODUCTION

One of the most common treatment strategies for brain metastasis (BM) is stereotactic radiosurgery (SRS) or fractionated SRS (FSRS).^1^ Large BM >2 cm is typically treated with FSRS, staged SRS, pre‐operative SRS/FSRS, or post‐operative SRS/FSRS.^2^ Small BM can be managed with all of these options as well but generally SRS is preferred.^2^

Leksell Gamma Knife^®^ (LGK, Elekta AB, Stockholm, Sweden) is a well‐established radiosurgery device for the treatment of intracranial lesions. The Perfexion^TM^, introduced in 2006, contains 192 ^60^Co sources focused at the unit center point.^3^ Eight motorized sectors, each containing 24 sources, can move over a tungsten collimation ring system providing three collimations of 4‐, 8‐ or 16‐mm diameter.^4^ The subsequent generation, Icon^TM^, introduced in 2015, uses the same delivery and patient positioning system as the Perfexion but offers additional systems such as a kilovoltage (kV) cone‐beam computed tomography (CBCT) imaging arm and an intra‐fraction motion management (IFMM) system.^5^ The CBCT is used for stereotactic coordinate definition or target localization.^5^ The IFMM system using infrared light monitors intrafraction motion.^5^ The latest generation, the Esprit, introduced in 2022, improves on the Icon with a more streamlined user interface, improved couch/vault door integration and faster treatment times (Figure 1(a)). Besides the historical G‐frame, the new Leksell^®^ Vantage^TM^ head frame, which was designed to significantly increase patient comfort, is available for all LGK units (Perfexion or later generations).^6^ For Icon and Esprit units, a thermoplastic mask is additionally available for SRS or FSRS.^4^

**FIGURE 1:**
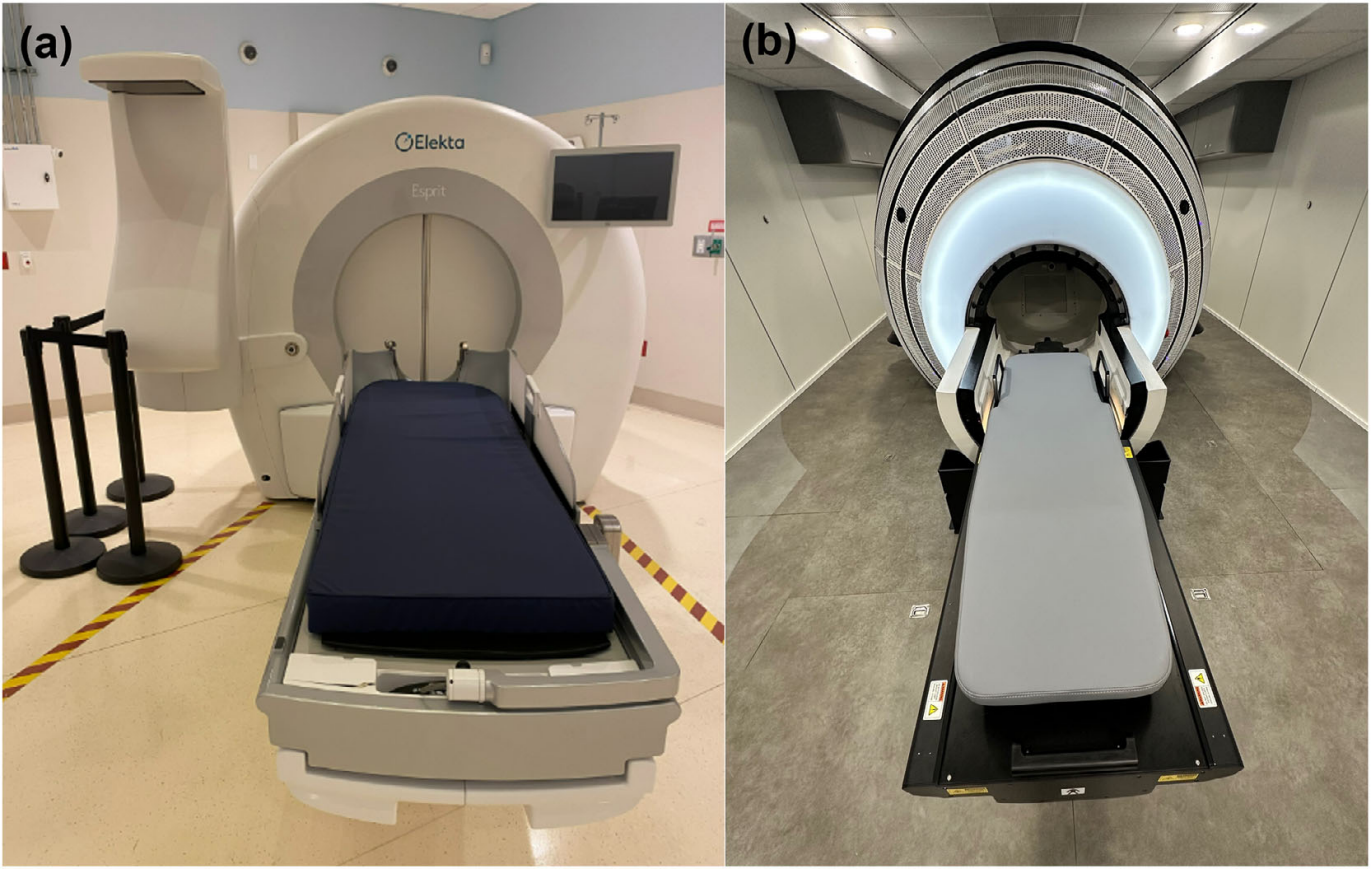
(a) Leksell Gamma Knife Esprit unit and (b) ZAP‐X unit.

Leksell GammaPlan^®^ (LGP, Elekta AB) has evolved over decades. Historically, manual forward planning was the primary planning approach. In 2010, an inverse planning tool using non‐convex optimization was introduced in LGP 10.^7^ In 2020, a novel inverse planning optimizer called fast inverse planning, commercially referred to as Lightning, was released.^7^ The Lightning dose optimizer utilizes sector‐duration optimization (convex) using linear programing to enhance planning efficiency.^7^ During dose optimization, Lightning carries out three sequential steps: (1) Isocenter placement: isocenters are chosen in the target; (2) Optimization: inverse planning is performed based on objectives including maximum dose to the target and organs‐at‐risk (OARs) as well as beam‐on‐time (BOT) penalization to determine times for each sector and collimator; (3) Sequencing: these times are converted into deliverable shots. Previous studies demonstrated that Lighting promptly generates plans which require minimal to no manual adjustment and there is minimal inter‐planner plan quality variability regardless of planner's experience.^8^, ^9^, ^10^, ^11^ For dose calculations, LGP uses a tissue‐maximum‐ratio (TMR) based algorithm, that is, TMR 10, which models the head as water and does not account for heterogeneity.^12^ Version 11.0.3 or later additionally offers a convolution algorithm which has not yet been well adopted clinically.

The ZAP‐X^®^ Gyroscopic Radiosurgery^®^ platform (Zap Surgical Systems, Inc., San Carlos, CA) is a novel radiosurgery device developed to treat intracranial and cervical spine lesions (Figure 1(b)). The system design, consisting of a shielded unit, table shielding (pneumatic door and rotary shell) and rotating beam stop, creates a self‐shielding effect during beam delivery and therefore, obviates the need for a shielded vault.^13^ A linear accelerator (linac), which is mounted on coupled gimbals, moves in dual axes of independent rotations about the isocenter and produces a wide range of non‐coplanar beams (3 megavoltage [MV], flattening filter free) covering a solid angle of more than 2π steradians.^13^ The collimator wheel attached to the front of the linac rotates to achieve circular collimations of 4‐, 5‐, 7.5‐, 10‐, 12.5‐, 15‐, 20‐, or 25‐mm diameter at a source to axis distance (SAD) of 45 cm.^14^ Couch motions are limited to three degrees of freedom (yaw: left‐right; longitudinal: superior‐inferior; pitch: anterior‐posterior) to align planned isocenters (similar to shots in LGP) to the machine isocenter.^14^, ^15^ An internally mounted kV imaging system, comprising a single x‐ray tube and a single imager, is used for pretreatment localization and intrafraction motion monitoring.^13^, ^14^ Immobilization is achieved with a mask, and an additional add‐on system, PinZ immobilizer with pins, is available in the DP‐1012 release. Table 1 summarizes the characteristics of the LGK and ZAP‐X devices.

**TABLE 1:** Overview of treatment units and treatment planning systems for Leksell Gamma Knife^®^ and ZAP‐X.

	Treatment unit	
	Leksell Gamma Knife^®^	ZAP‐X
Beam energy	Avg. 1.25 MeV (Co‐60)	3 MV FFF
SAD (cm)	37.4–43.3	45
Collimator size (mm)	4, 8 and 16 (Perfexion or later generations)	4, 5, 7.5, 10, 15, 20 and 25
Delivery technique	Non‐coplanar	Non‐coplanar
Imaging system	kV CBCT (Icon or Esprit)	Planar kV
Immobilization	Mask, frame	Mask, PinZ immobilizer

	Treatment planning system	
	LGK Lightning	ZAP‐Axon Krylov
Dose calculation algorithm	TMR 10 (heterogeneity uncorrected)	Analytic Ray Tracing (heterogeneity corrected)
Planning algorithm	Linear convex	Linear convex
Cost function	Linear	Quadratic
Solving method	Direct	Iterative

Abbreviations: CBCT, cone beam computed tomography; FFF, flattening filter free; kV, kilovoltage; LGK, Leksell Gamma Knife; MeV, mega electron voltage; MV, megavoltage; SAD, source‐to‐axis distance; TMR, tissue maximum ratio.

The ZAP‐X has a dedicated treatment planning system (TPS) and its TPS has evolved over the past few years. In its initial TPS version (refer to as Legacy TPS), isocenters are manually placed first and the optimizer runs with proper settings. In the DP‐1010/1011 releases, the Advanced Workspace became available as an option within Legacy TPS. While both Legacy and Advanced have the same optimization algorithm (i.e., direct inversions solve the convex problem) to minimize the linear cost function, the Advanced Workspace offers an automated isocenter placement tool and a more robust implementation of the optimization algorithm to handle more candidate beams. A recently launched new TPS application, named ZAP‐Axon^®^, deploys a new optimization algorithm (Krylov optimizer) that uses a quadratic cost function and handles even more beams than the Advanced Workspace. Because the Krylov optimizer leverages an iterative solver to address the convex problem, the optimization time is faster than older versions (Legacy TPS and Advanced Workspace). For dose calculations, ZAP‐X TPS uses an Analytic Ray Tracing algorithm which accounts for heterogeneity based on one‐dimensional equivalent path length scaling.^13^ Table 1 compares the LGK Lightning and ZAP‐Axon Krylov algorithms.

While several treatment planning studies using the LGK Lightning dose optimizer have been published,^9^, ^10^, ^11^, ^16^ to the best of our knowledge, similar studies using ZAP‐Axon have not yet been published. In this study, SRS treatment plan quality between ZAP‐Axon Krylov optimizer and LGK Lighting optimizer is compared for single brain metastatic lesions. Both large BM (post‐operative resection cavities and intact) and small BM (intact) are included in this study.

## 2 METHODS

### 2.1 Patient cohort

Thirty BM patients (*n* = 30) who were previously treated with LGK SRS or FSRS were selected and were divided into three groups (10 patients per group). The first group (BM_lg, postop_) consisted of post‐operative resection cavities of BM and received FSRS to a planning target volume (PTV) with a regimen of 27 Gy in three fractions per our institutional paradigm.^2^ The clinical target volume was derived from a 2‐mm uniform expansion of the gross target volume (GTV). No setup margin was used to derive the PTV. The second group (BM_lg, intact_) had a single, large intact BM and received staged SRS to the GTV to 15 Gy in one fraction per stage based on institutional dosing guidelines.^17^ For this group, the first of the 2‐stage treatment was chosen. The third group (BM_sm, intact_) consisted of a single, small intact BM and received SRS to the GTV to 24 Gy in one fraction. The lesions in all three groups were not adjacent (>4 cm) to OARs such as brainstem, optic chiasm or optic nerves. Table 2 shows lesion characteristics, number of lesions (patients), mean volume (range) of the lesions and prescription dose per group. This study was approved by our Institutional Review Board.

**TABLE 2:** Patient cohort selected for this study.

Group	BM_lg, postop_	BM_lg, intact_	BM_sm, intact_
Lesion characteristics	Large, post‐operative resection cavity of a brain metastasis	Large, intact brain metastasis	Small, intact brain metastasis
No. of lesions (patients)	10	10	10
Mean volume (range) (cm^3^)	14.67 (9.92–18.45)	5.20 (3.04–8.33)	0.11 (0.05–0.21)
Prescription dose	27 Gy in 3 fractions (FSRS) to PTV	15 Gy in 1 fraction (staged SRS) to GTV	24 Gy in 1 fraction (SRS) to GTV

Abbreviations: BM, brain metastasis; FSRS, fractionated stereotactic radiosurgery; GTV, gross tumor volume; SRS, stereotactic radiosurgery; PTV, planning target volume.

### 2.2 Treatment planning

The 30 clinical cases were replanned in ZAP‐Axon based on our institutional SRS/FSRS planning guidelines. High resolution brain computed tomography (CT) images, which were fused to planning magnetic resonance images used for LGK SRS/FSRS, and contours were imported in ZAP‐Axon. For treatment planning, two steps were executed. First, after proper parameter settings (Isocenter Count Limit, Path Density, Collimator Overlap, Small Target Diameter and Target Dilation), target packing ran for automated isocenter placement (Figure 2(a)). Second, the optimizer ran with relevant parameter settings (Interior‐100% Max, Interior‐100% Min, Ext. 1 mm Max, Ext. 2 mm Max, and Ext. 5 mm Max) (Figure 2(a)). Since OARs were not close (>4 cm) to targets, maximum dose to OARs were not included in the optimizer. Default target coverage (TC) was set at 99.5%. Before optimization, the Krylov optimizer was enabled. To minimize planner dependence on planning, no manual adjustments were made after optimization was completed; in other words, no isocenter was moved, added, or deleted, no collimator size was manually changed, or no dose was scaled. The two steps were repeated until TC, Paddick conformity index (PCI), treatment time, and gradient index (GI) were achieved in order of this importance. Once planning goals were met or close and isodose distributions looked clinically acceptable, the optimizer was not rerun, and the last plan was chosen for plan comparison. Table 3 describes each parameter for isocenter placement and for the optimizer in ZAP‐Axon. Our institutional SRS/FSRS planning guidelines include TC ≥99.5% receiving prescription dose or higher, PCI as close to 1.0 as possible, GI ≤3.0 and a prescription isodose line (Rx IDL) ≥50% and ≤93%. PCI and GI are defined according to methods described by Paddick.^18^, ^19^

**FIGURE 2:**
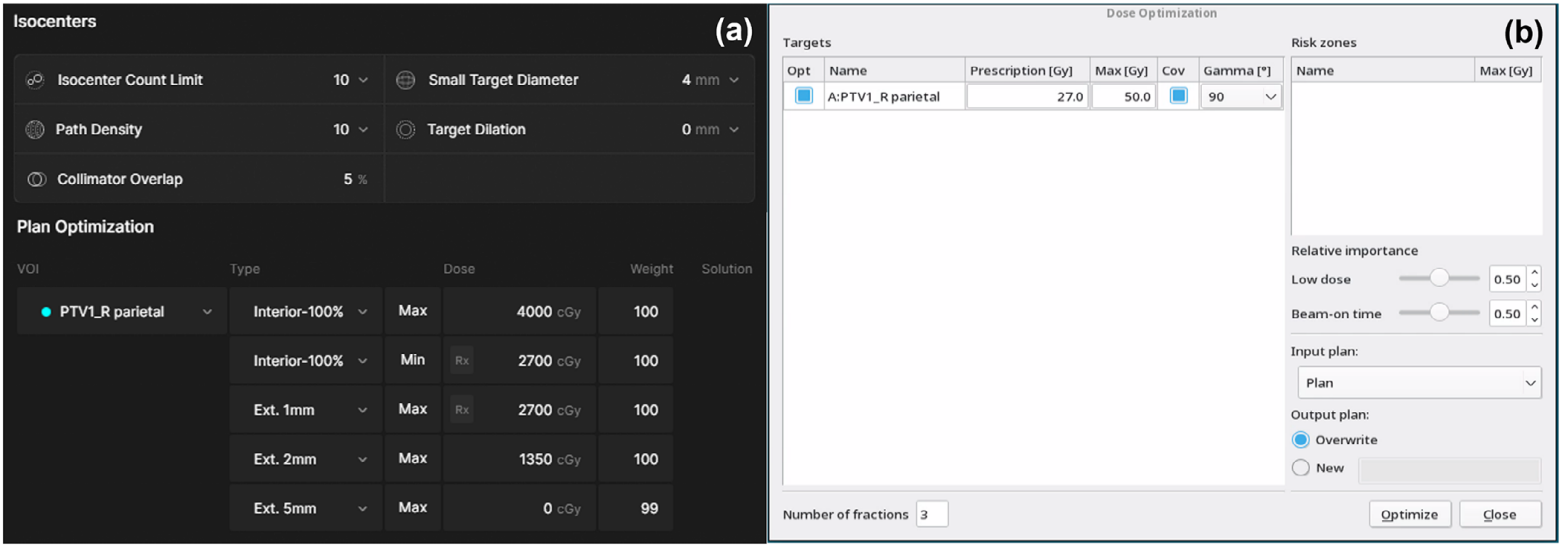
Examples of parameter settings for (a) ZAP‐Axon Krylov optimizer and (b) LGK Lightning optimizer for a post‐operative resection cavity of brain metastasis.

**TABLE 3:** Description for parameters in ZAP‐Axon Krylov optimizer and Leksell Gamma Knife Lightning optimizer.

ZAP‐Axon Krylov	Isocenter placement	Isocenter count limit: 0–75, no limit	Maximum number of spheres (isocenters and collimators) considered for packing each target
		Small target diameter: 4–10 mm	Targets smaller than this minimum diameter are not sphere packed; they receive a single sphere with its isocenter on the target's center of mass
		Path density: 3–17	Number of candidate beam paths considered in the treatment plan optimization
		Target dilation: 0–2 mm	Dilation shell added to the target's volume that will be packed with spheres
		Collimator overlap: −200–75%	Maximum overlap allowed between spheres during the packing optimization step
	Optimizer	Interior‐100%, Max (cGy)	Maximum dose limit to 100% of voxels within the target
		Interior‐100%, Min (cGy)	Minimum dose limit to 100% of voxels within the target
		Ext. 1 mm, Max (cGy)	Maximum dose limit to points on the surface of a 1 mm shell around the target
		Ext. 2 mm, Max (cGy)	Maximum dose limit to points on the surface of a 2 mm shell around the target
		Ext. 5 mm, Max (cGy)	Maximum dose limit to points on the surface of a 5 mm shell around the target
Leksell Gamma Knife Lightning	Optimizer	Low dose: 0.00–1.00	Relative importance of minimizing the overall low dose volume to surrounding tissue outside the target
		Beam‐on time: 0.00–1.00	Relative importance of minimizing the beam‐on time
		Max (Gy)	Maximum dose limit to the target
		Cov (%)	If enabled, the target coverage to 99.0% or more increases

The 30 cases were also replanned in LGP (v.11.4.2) using the Lightning optimizer. To keep the same image resolution as in ZAP‐Axon plans, CT images and contours used in ZAP‐Axon were imported in LGP. In the dose optimizer (Figure 2(b)), prescription dose, proper maximum dose to the target, number of fractions (one or three, only for mask fixation configuration) were entered and the TC box was enabled. Low dose (LD) and BOT settings ranging from 0.00 to 1.00 each were selected. Like in the Krylov optimizer, maximum dose to OARs were not included in the Lighting optimizer. The optimizer ran repeatedly with different LD/BOT combinations until TC, PCI, BOT and GI were achieved in order of this importance. No manual adjustments were made after optimization completion. Once planning goals were met or close and isodose distributions looked clinically acceptable, the final plan was chosen for plan comparison. Table 3 defines each parameter for the Lightning optimizer.

### 2.3 Comparison of plan quality metrics between two optimizers

Plan quality metrics were noted for each group. For Krylov plans, PCI, GI, TC (%), Rx IDL (%), treatment time (= gantry motion + beam‐on + intrafraction imaging) (min), number of isocenters, and number of beams were recorded. For Lightning plans, PCI, GI, TC (%), Rx IDL (%), sBOT (scaled BOT to dose rate of 3.0 Gy/min) (min) and number of shots were recorded. Parameter settings for each optimizer were also noted.

Statistical analysis was performed to compare plan quality metrics between Krylov plans and Lightning plans for each group. Considering small sample size (*n* = 10) per group, a Wilcoxon matched pairs test was performed. A *p‐value* <0.05 was considered statistically significant.

## 3 RESULTS

### 3.1 Plan quality metrics

Table 4 summarizes comparison of plan quality metrics between Krylov plans and Lightning plans. For the BM_lg, postop_ group, mean values of PCI, GI, TC (%), Rx IDL (%), and treatment time/sBOT (min) for Krylov versus Lightning plans were 0.91 versus 0.91, 2.48 versus 2.56, 99.5% versus 99.7%, 51.1% vs, 53.7% and 37.4 min versus 36.5 min, respectively. All metrics except for Rx IDL (%) were not statistically significantly different. For the BM_lg, intact_ group, corresponding values were 0.93 versus 0.93, 2.42 versus 2.57, 99.5% versus 99.7%. 51.5% versus 53.0% and 35.9 min versus 36.3 min. While GI was statistically significantly different (*p* <0.05) between the two optimizers, the rest of the metrics were not. For the BM_sm, intact_ group, corresponding values were 0.71 versus 0.64, 3.10 versus 3.26, 99.5% versus 99.5%, 61.9% versus 53.0% and 8.1 min versus 19.8 min. In this group, PCI and treatment time/sBOT were statistically significantly different (*p* <0.05), whereas GI and Rx IDL were not. For TC (%), statistical analysis was not performed because all Krylov plans had the same TC (99.5%). Figure 3 plots plan quality metrics (PCI (a)‐(c), GI (d)‐(f), TC (g)‐(i), Rx IDL (j)‐(l), and treatment time or sBOT (m)‐(o)). In each figure, Box and Whisker plots from left to right represent ZAP‐Axon Krylov, LGK Lightning and clinical LGK plans in order. Each plot displays lower and upper quartiles (box), median (horizontal line inside the box), mean (marker × inside the box), minimum and maximum values (two lines outside the box) and outliers (s) (dot[s]) of the data. Examples of isocenter and shot placement and examples of ZAP‐Axon Krylov and LGK Lightning plans for each group are shown in Figures 4 and 5, respectively.

**TABLE 4:** Plan quality metrics (mean [range]) comparison between ZAP‐Axon Krylov plans and Leksell Gamma Knife Lightning plans.

Group	BM_lg, postop_		BM_lg, intact_		BM_sm, intact_	
	ZAP‐Axon Krylov	LGK Lightning	ZAP‐Axon Krylov	LGK Lightning	ZAP‐Axon Krylov	LGK Lightning
PCI	0.91 (0.89–0.93)	0.91 (0.89–0.94)	0.93 (0.91–0.94)	0.93 (0.91–0.96)	0.71 (0.42–0.89)	0.64 (0.42–0.86)
	*P* = 0.8125		*P* = 0.5781		*P* <0.05	
GI	2.48 (2.17–2.79)	2.56 (2.37–2.77)	2.42 (2.28–2.54)	2.57 (2.49–2.65)	3.10 (2.87–3.45)	3.27 (2.86–3.56)
	*P* = 0.2754		*P* <0.05		*P* = 0.1602	
TC (%)	99.5 (99.5–99.5)	99.7 (99.5–99.8)	99.5 (99.5–99.5)	99.7 (99.5–99.9)	99.5 (99.5–99.5)	99.5 (99.5–99.6)
	N/A		N/A		N/A	
Rx IDL (%)	51.1 (50.0–52.6)	53.7 (53.0–55.0)	51.5 (50.0–55.2)	53.0 (52.0–54.0)	61.9 (50.7–77.6)	53.0 (50.0–63.0)
	*P* <0.05		*P* = 0.1934		*P* = 0.0547	
Tx time or sBOT (min)	37.4 (27.0–50.0)	36.5 (25.1–48.1)	35.9 (33.0–40.0)	36.3 (28.7–46.6)	8.1 (5.0–11.0)	19.8 (15.0–25.8)
	*P* = 0.4992		*P* = 0.7695		*P* <0.05	
Number of isocenters or shots	11.2 (8–15)	58.7 (41–84)	8.4 (8–10)	40.8 (32–51)	1 (1–1)	4.6 (3–7)
Number of beams	283.8 (232–377)	N/A	316.5 (234–382)	N/A	64 (37–111)	N/A

Abbreviations: BM_lg, postop_, large post‐operative resection cavity of brain metastasis; BM_lg, intact_, large intact brain metastasis; BM_sm, intact_, small intact brain metastasis; GI, gradient index; LGK, Leksell Gamma Knife; PCI, Paddick conformity index; Rx IDL, prescription isodose line; sBOT, scaled beam‐on time; TC, target coverage; Tx, treatment

**FIGURE 3:**
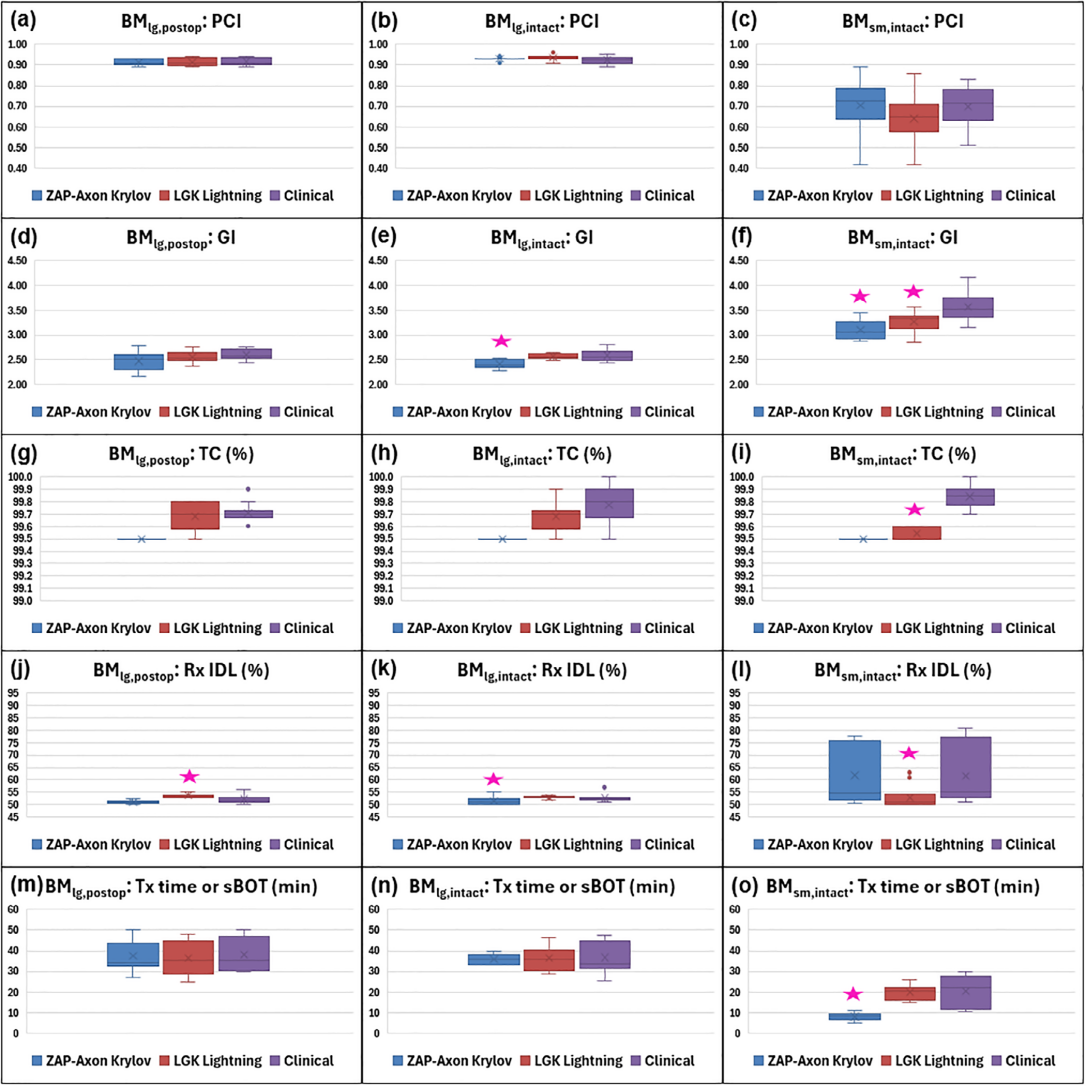
(a)–(o) Comparison of plan quality metrics among ZAP‐Axon Krylov plans, LGK Lightning plans and clinical LGK plans. Pink stars represent the Krylov or Lightning plans whose plan quality metrics are statistically significantly different from those of clinical plans. Abbreviations: BM_lg, postop_, large post‐operative resection cavity of brain metastasis; BM_lg, intact_, large intact brain metastasis; BM_sm, intact_, small intact brain metastasis; GI, gradient index; LGK, Leksell Gamma Knife; PCI, Paddick conformity index; Rx IDL, prescription isodose line; sBOT, scaled beam‐on time; TC, target coverage; Tx, treatment.

**FIGURE 4:**
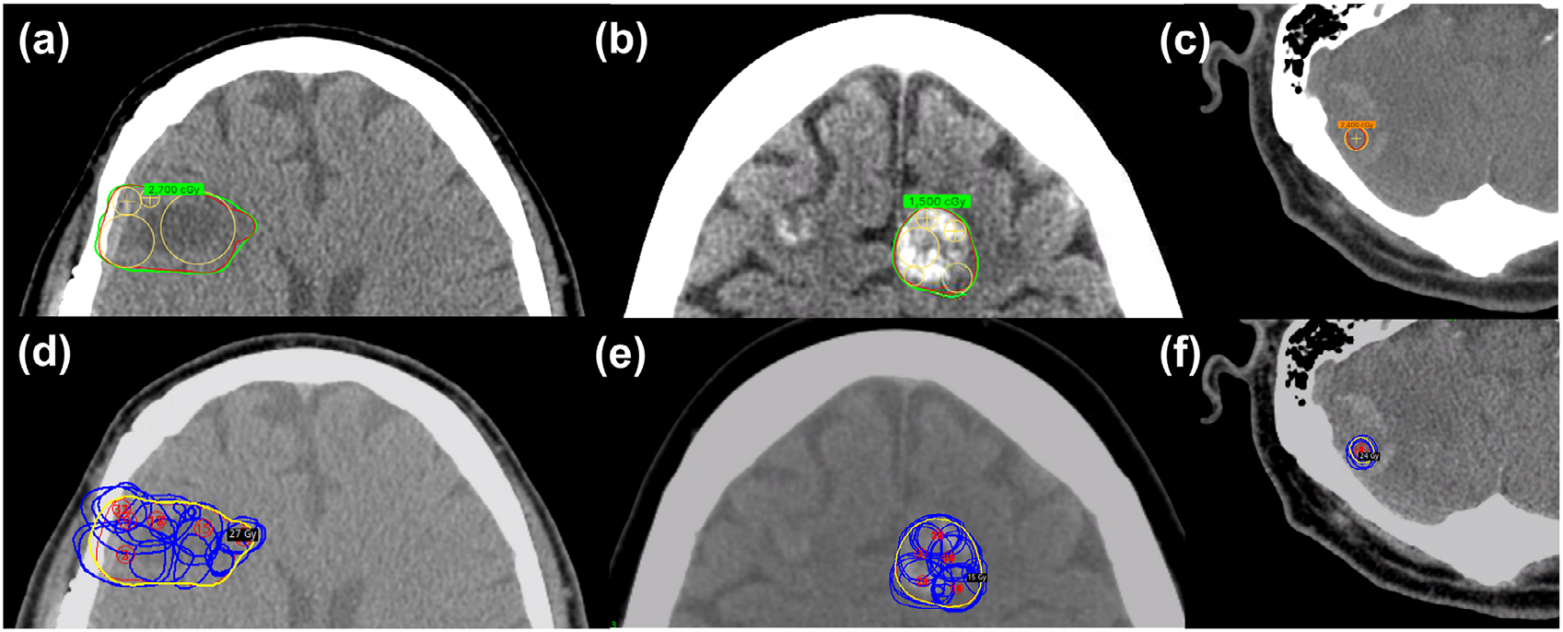
Representative examples of (a)–(c) isocenter placement in ZAP‐Axon Krylov plans and (d)–(f) shot placement in LGK Lightning plans for BM_lg, postop_, BM_lg, intact_, and BM_sm, intact_ groups. Abbreviations: BM_lg, postop_, large post‐operative resection cavity of brain metastasis; BM_lg, intact_, large intact brain metastasis; BM_sm, intact_, small intact brain metastasis; LGK, Leksell Gamma Knife.

**FIGURE 5:**
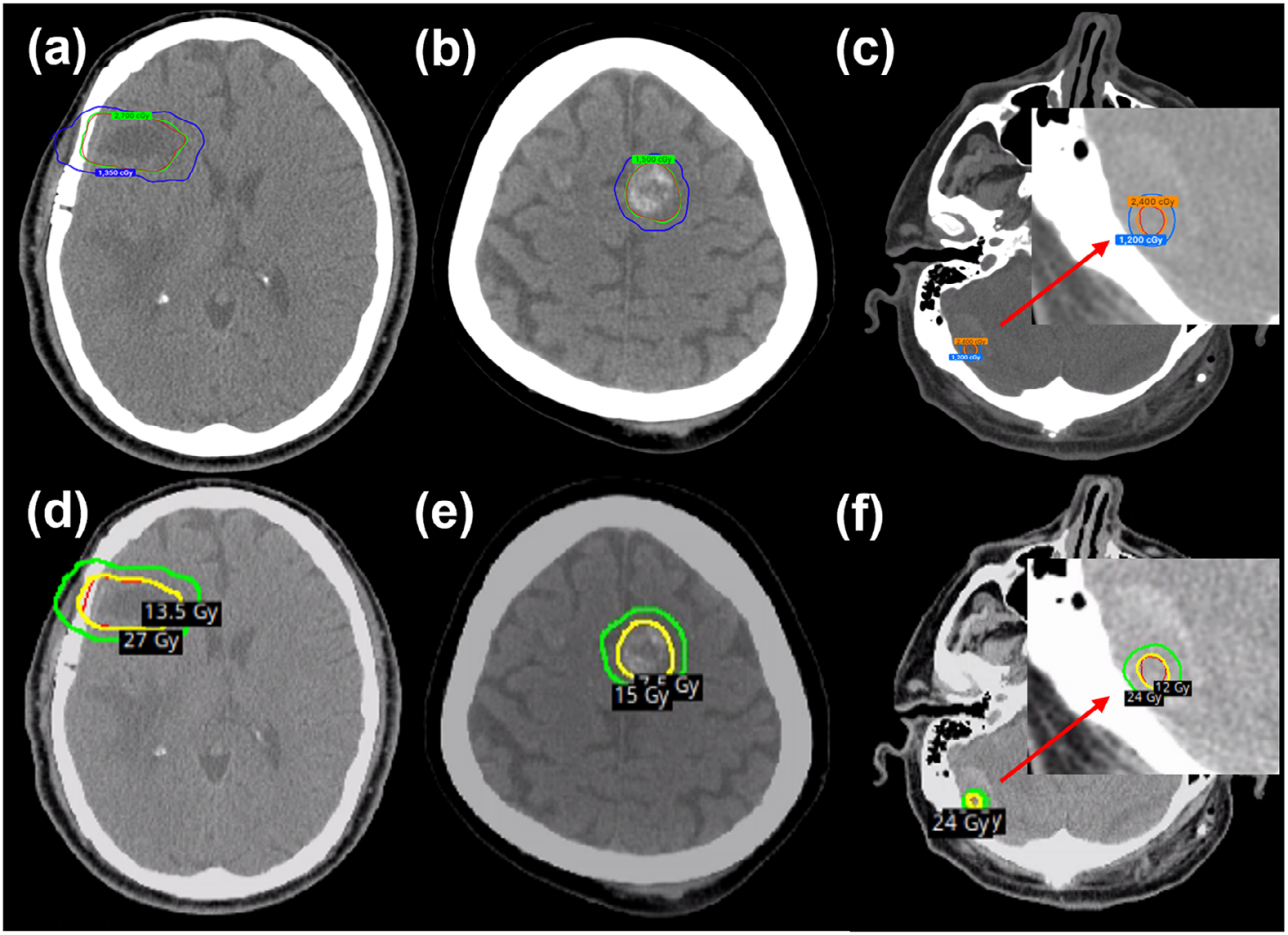
Axial views of representative examples of (a)–(c) ZAP‐Axon Krylov plans and (d)–(f) LGK Lightning plans for BM_lg, postop_, BM_lg, intact_, and BM_sm, intact_ groups. Abbreviations: BM_lg, postop_, large post‐operative resection cavity of brain metastasis; BM_lg, intact_, large intact brain metastasis; BM_sm, intact_, small intact brain metastasis; LGK, Leksell Gamma Knife.

### 3.2 Optimizer parameter settings

Table 5 presents parameter settings used in ZAP‐Axon. Parameters for isocenter placement were chosen as follows: Isocenter count limit ranged from 8 to 15 and from 8 to 10 for BM_lg, postop_ and BM_lg, intact_ groups, respectively but “no limit” was selected for the BM_sm, intact_ group. For small target diameter, 4 mm was selected for two large BM groups and 4, 5, and 7.5 mm were selected for the small BM group. Path densities of 10 ‐ 15, 10 ‐ 15 and 6 were chosen for the three groups in order. Target dilation of 0 mm was kept for all 30 plans. Collimator overlaps of 5%, 10% and 10% were selected for BM_lg, postop_, BM_lg, intact_, and BM_sm, intact_ groups, respectively. For Krylov optimization parameter settings, Interor−100% max, Interior−100% min, Ext. 1 mm max, Ext. 2 mm max and Ext. 5 mm max were set to prescription dose multiplied by 1.3 − 1.8, prescription dose, prescription dose, prescription dose/2 and 0, respectively, for all three groups.

**TABLE 5:** Summary of parameter settings (range) in ZAP‐Axon Krylov optimizer and Leksell Gamma Knife Lightning optimizer.

			Group		
	Optimizer parameters		BM_lg, postop_	BM_lg, intact_	BM_sm, intact_
ZAP‐Axon Krylov	Isocenter placement	Isocenter count limit	8–15	8–10	No limit
		Small target diameter (mm)	4	4	4, 5 or 7.5
		Path density	10–15	10–15	6
		Target dilation (mm)	0	0	0
		Collimator overlap (%)	5	10	10
	Optimizer	Interior‐100%, Max (Gy)	35.0–40.0	19.0–20.0	42.0–43.2
		Interior‐100%, Min (Gy)	27.0	15.0	24.0
		Ext. 1 mm, Max (Gy)	27.0	15.0	24.0
		Ext. 2 mm, Max (Gy)	13.5	7.5	12.0
		Ext. 5 mm, Max (Gy)	0.0	0.0	0.0
Leksell Gamma Knife Lightning	Optimizer	Low dose	0.50–0.53	0.48–0.51	0.50–0.80
		Beam‐on time	0.48–0.50	0.48–0.56	0.40–0.50
		Max (Gy)	50.0	27.8	38.0–43.2
		Cov (%)	Enabled	Enabled	Enabled

Abbreviations: BM_lg, postop_, large post‐operative resection cavity of brain metastasis; BM_lg, intact_, large intact brain metastasis; BM_sm, intact_, small intact brain metastasis; Cov, coverage

LGK Lightning optimizer settings are also presented in Table 5. LD was selected in the range of 0.50 – 0.53, 0.48 – 0.51 and 0.50 ‐ 0.80 for BM_lg, postop_, BM_lg, intact_, and BM_sm, intact_ groups, respectively. Corresponding ranges for BOT were 0.48 – 0.50, 0.48 – 0.56 and 0.40 – 0.50. For max dose to the target, prescription dose multiplied by 1.6 – 1.9 was entered.

## 4 DISCUSSION

In this study, SRS treatment plan quality between the ZAP‐Axon Krylov optimizer and the LGK Lighting optimizer was assessed to quantify plan quality differences. For the cases evaluated, PCI for Krylov plans was comparable to or better than that for Lightning plans. While mean PCI was similar between the two optimizers for both large BM groups, Krylov outperformed in achieving greater PCI for the small BM group (Table 4). This is attributed to Lightning plans’ using multiple shots (mean number of shots: 4.6) with 8‐mm and 16‐mm sector collimations for small lesions. Plan quality was not improved much with greater LD (0.80) and smaller BOT settings (0.40). The plan quality difference became larger when the target was elongated in the superior‐inferior direction (Figure 6). On the other hand, mean PCI was similar between Krylov plans and clinical LGK plans (0.71 vs. 0.70, *p* = 0.9102) (Figure 3(c)). Clinical cases with small targets are typically adjusted manually after Lightning optimizer runs or they are planned manually to achieve better conformality.

**FIGURE 6:**
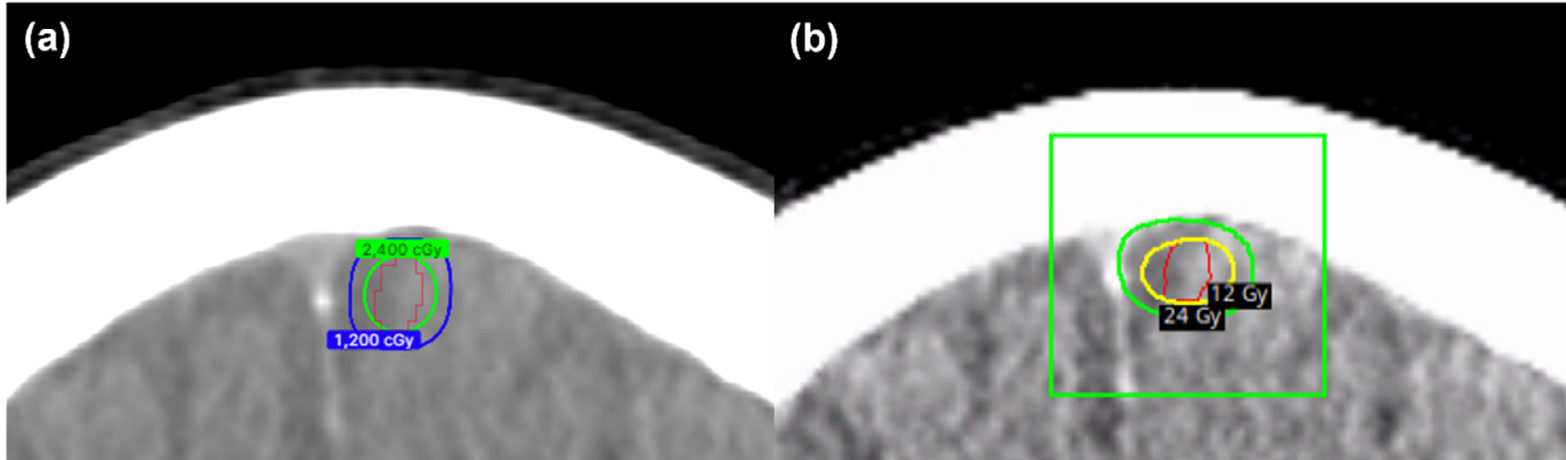
Coronal views of an example of (a) ZAP‐Axon Krylov plan and (b) LGK Lightning plan for BM_sm, intact_ group. Abbreviations: BM_sm, intact_, small intact brain metastasis; LGK, Leksell Gamma Knife.

Regardless of statistical analysis results, the Krylov optimizer achieved smaller GI than Lightning for all three groups (Table 4). In the Krylov optimizer, a quadratic cost function is minimized using multi‐criteria optimization which focuses on both PCI and GI. The Krylov optimizer achieved the smallest mean GI for the BM_lg, intact_ group because of the target size and shape (Figure 4(b)). For the BM_sm, intact_ group, with a single isocenter, smaller GI (mean GI: 3.10) was achieved using multiple non‐coplanar beams (mean number of beams: 64). In contrast, Lightning used multiple shots (mean number of shots: 4.6) which usually help create conformal beam shapes using sector‐duration optimization (Figure 4(f)). For GI, the Lightning optimizer typically outperforms manual forward planning or a combination of Lightning and manual adjustments (typical clinical plans). This was also observed in this study as shown in Figure 3(f).

TC was better with Lightning plans for both large BM groups and comparable between the two optimizers for small BM lesions. In ZAP‐Axon, minimum TC was set at 99.5% as a default following our institutional planning guidelines. With a higher TC setting, higher TC could be achieved. Even though Lightning created plans with better TC for large BM lesions, the difference is unlikely to be of clinical significance. For small BM lesions, mean TC for clinical LGK plans was greater (99.8%) than both optimizer plans (Figure 3(i)) because our clinical practice is to cover small targets close to 100% especially with mask immobilization.

For both large BM groups, Rx IDL (%) for Krylov plans was slightly lower than that for Lightning plans. This would be due to the dilation selection and max dose setting. The 0‐mm dilation was selected to achieve greater PCI, resulting in lower Rx IDL, often <50%. In that scenario, the optimizer re‐ran with a smaller max dose setting to achieve Rx IDL >50%. The Rx IDL difference between the two optimizers would be clinically minimal. For the small BM group, Rx IDL was higher for Krylov plans. In most Krylov plans, collimator size (4 mm ‐ 7.5 mm) was selected such that collimator covers the target enough, resulting in higher mean Rx IDL (61.9%).

Treatment time (= gantry motion + beam‐on + intrafraction imaging) for Krylov plans was compared with sBOT for Lightning plans. For the comparison, 10‐min automated initial alignment time, which is included in treatment time, was excluded because sBOT for Lightning plans does not include pre‐treatment imaging time. sBOT does not include time for table motions between shots which can take from 2 min to 4 min depending on the total number of shots. For the two large BM groups, Krylov plans were generated such that treatment time was similar to sBOT for Lightning plans by having adequate number of isocenters. For small BM lesions, Krylov plans had shorter treatment time because target volume was small (mean GTV volume: 0.11 cm^3^) enough to have a single isocenter and small number of beams (mean number of beams: 64). Treatment time usually increases with increasing isocenters in ZAP‐Axon plans as imaging time between isocenters and intrafraction imaging time are additionally added. On the other hand, sBOT was comparable between Lightning plans and clinical LGK plans (mean sBOT: 19.8 min vs. 20.7 min, *p* = 0.6250) (Figure 3(o)).

For ZAP‐Axon plans, parameter settings for isocenter placement can affect plan quality. As stated above, smaller target dilation (e.g., 0 mm: collimator edge will not be outside the target) with the same number of isocenters can improve PCI but lower Rx IDL (i.e., increase hot spot). Isocenter count limit can determine maximum number of isocenters and affect treatment time. Including smaller collimator(s) (small target diameter) can also improve PCI but increases number of MUs and thereby, results in increased treatment time. Higher path density (e.g., 15) can improve PCI but in some cases, the optimizer does not allow for optimization because of beam path(s) falling outside the clearance zone. Large collimator overlap (>30%) can increase hot spot or can place small number of isocenters with larger collimators, leading to compromised plan quality.

Figure 7 shows the number of iterations and optimization time per iteration for each optimizer plans. Mean numbers of iterations, which were run for Krylov versus Lighting plans, were 2.5 versus 2.0, 2.1 versus 2.2 and 2.8 versus 2.3 for BM_lg, postop_, BM_lg, intact_, and BM_sm, intact_ groups, respectively (Figure 7(a)–(c)). Mean optimization times per iteration for the final Krylov versus Lighting plans were 24.5 min versus 1.4 min, 298.6 sec versus 27.9 sec and 51.9 sec versus 7.4 sec for the three groups in order (Figure 7(d)–(f)). Optimization time per iteration was significantly longer in ZAP‐Axon TPS. One of the contributing factors was dose calculation grid size: In ZAP‐Axon, dose calculation grid resolution in the entire CT dataset is the same as CT resolution (∼0.5 mm × 0.5 mm × 1.0 mm), whereas in LGP, grid resolution is 0.5 mm only within the box around the target with extra fine enabled as seen in Figure 6(b) and 4 mm outside the box.

**FIGURE 7:**
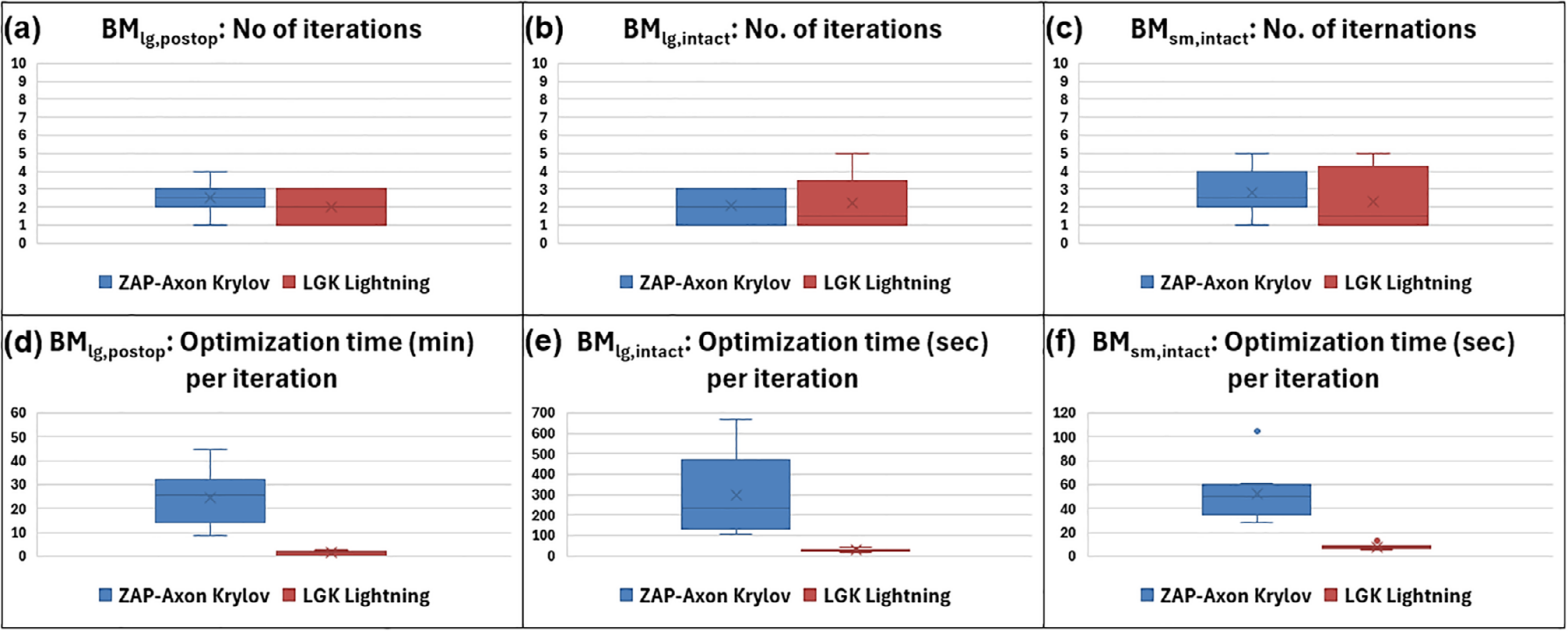
(a)–(c) Number of iterations and (d)–(f) optimization time per iteration for ZAP‐Axon Krylov plans and LGK Lightning plans for BM_lg, postop_, BM_lg, intact_, and BM_sm, intact_ groups. Abbreviations: BM_lg, postop_, large post‐operative resection cavity of brain metastasis; BM_lg, intact_, large intact brain metastasis; BM_sm, intact_, small intact brain metastasis; LGK, Leksell Gamma Knife.

Besides planning algorithms, observed plan quality differences between ZAP‐Axon Krylov and LGK Lighting plans may be attributable to machine design. Both machines have similar energies (avg. 1.25 MeV for LGK versus 3 MV [mean energy ∼1 MV] for ZAP‐X), similar SADs (37.4–43.3 cm for LGK versus 45 cm for ZAP‐X), collimator‐based design and isocentric delivery by table motion with non‐coplanar beams (Table 1). However, collimator configuration and collimator size options are different between the two machines. LGK has eight sectors (24 sources per sector, Perfexion or later generations) in a fixed hemispherical helmet, and each sector can move independently of the others and set to one of the four different positions (blocked, 4 mm, 8 mm or 16 mm).^8^ Per shot (beam), up to 192 sources simultaneously converse at the unit center point.^3^ This sector configuration produces complex shot dose distributions.^8^ In contrast, ZAP‐X delivers step‐and‐shoot collimated beams isocentrically and per isocenter with one selected collimator size (4 mm, 5 mm, 7.5 mm, 10 mm, 12.5 mm, 15 mm, 20 mm or 25 mm), a single beam to multiple beams can be delivered.^20^ Because of the unique collimator configuration of LGK, the maximum solid angle covered by the source is 1.25 πR^2^ which gives fixed beam angles, whereas the corresponding angle for ZAP‐X is 2.83 πR^2^, which allows for a maximum of 1860 non‐coplanar beams and consequently, helps improve plan quality (especially PCI).^21^ Although the penumbra width of a ZAP‐X beam is comparable to that of a LGK beam (1‐2 mm),^22^, ^23^ final isodose distributions created by multiple shots (LGK) or multiple isocenters/beams (ZAP‐X) determine the beam penumbra, ultimately affecting GI.

There are several limitations to this study. 1) The dataset for this study was comprised of a small cohort (10 patients per group). 2) Only a single lesion was assessed. The optimizers can perform in a different manner when multiple lesions are optimized simultaneously. 3) Lesions abutting or near OARs were not investigated. When OARs are close to targets, dose constraints for OARs are included in the Lightning optimizer or Krylov optimizer and our institutional TC constraint (≥99.5%) is often not achieved, requiring manual adjustments. To avoid this scenario, lesions, not close to OARs, were chosen in this study. 4) Plan qualities involving expert manual adjustments and total planning time were not evaluated. Further studies and investigation will be warranted in the future.

## 5 CONCLUSIONS

Without any manual adjustments, ZAP‐Axon Krylov plans were comparable to LGK Lightning plans for large BM lesions (volumes ranging from 3.04 cm^3^ to 18.45 cm^3^) but the Krylov optimizer outperformed the Lightning optimizer for small BM lesions (volumes ranging from 0.05 cm^3^ to 0.21 cm^3^). Overall, GI was better in Krylov plans and planning was more efficient with the Lightning optimizer. In ZAP‐Axon, parameter settings for isocenter placement and dose optimizer can affect plan quality markedly.

## AUTHOR CONTRIBUTIONS

Yongsook C. Lee designed the concept and wrote the initial version of the manuscript. Alexander Maslowski helped with ZAP‐Axon optimizer settings. Alexander Maslowski, Rupesh Kotecha, Ranjini Tolakanahalli, D Jay Wieczorek, Matthew D. Hall, Minesh P. Mehta, Michael W. McDermott, and Alonso N. Gutierrez provided scientific reviews and contributed to the final manuscript.

## CONFLICT OF INTEREST STATEMENT

Yongsook C. Lee received speaker honorarium from ZAP Surgical Systems, Inc. Alexander Maslowski is employed by ZAP Surgical Systems, Inc. Rupesh Kotecha received personal fees from Accuray, Inc., Elekta AB, ViewRay, Inc., Novacure, Inc., Elsevier, Inc., Brainlab, Kazia Therapeutics and Castle Biosciences, Inc. and received institutional research funding from Medtronic, Inc., Blue Earth Diagnostics Ltd., Novacure, Inc., GT Medical Technologies, AstraZeneca, Exelixis, ViewRay, Inc., Brainlab, Cantex Pharmaceuticals and Kazia Therapeutics. Matthew D. Hall received grants 8LA04 and 22L01, Live Like Bella Pediatric Cancer Research Initiative, Florida Department of Health. Ranjini Tolakanahalli has an ownership interest (stock) in Siemens Healthineers. Minesh P. Mehta received consulting fees from Karyopharm Therapeutics, Sapience Therapeutics, ZAP Surgical Systems, Inc., Mevion Medical Systems, Xoft, Inc. and Kazia Therapeutics and is a board of directors in Oncoceutics, Inc. and has an ownership interest (stock) in Chimerix, Inc. Michael W. McDermott received consulting fees from Stryker and has an ownership interest (stock) in Diende Medical and ZAP Surgical Systems, Inc. Alonso N. Gutierrez received personal fees from Elekta AB, ViewRay, Inc., IBA AB and Accuray, Inc., and has an ownership interest in Atlantic Health Solutions (uPlanTM).

## ETHICAL APPROVAL

This study was approved by the institutional review board of Herbert Wertheim Cancer Institute, Baptist Health South Florida.

## Data Availability

Data and materials provided in this study will not be shared.
